# Assessment of MGMT and TERT Subtypes and Prognosis of Glioblastoma by Whole Tumor Apparent Diffusion Coefficient Histogram Analysis

**DOI:** 10.1002/brb3.70175

**Published:** 2024-12-31

**Authors:** Ling Chen, Min Wu, Yao Li, Lifang Tang, Chuyun Tang, Lizhao Huang, Tao Li, Li Zhu

**Affiliations:** ^1^ Department of Radiology Liuzhou Worker's Hospital Guangxi China; ^2^ Department of Neurosurgery Liuzhou Worker's Hospital Guangxi China; ^3^ Department of Radiology The First Affiliated Hospital of Guangxi Medical University Guangxi China

**Keywords:** apparent diffusion coefficient histogram, diffusion‐weighted imaging, glioblastoma, MGMT, TERT

## Abstract

**Background**: Adult glioblastomas (GBMs) are associated with high recurrence and mortality. Personalized treatment based on molecular markers may help improve the prognosis. We aimed to evaluate whether apparent diffusion coefficient (ADC) histogram analysis can better predict *MGMT* and *TERT* molecular characteristics and to determine the prognostic relevance of genetic profile in patients with GBM.

**Materials and Methods**: MRI, clinical, and pathological data of 79 patients with GBM were retrospectively collected. The ADC values based on histogram analysis were described using 10th percentile (p10), 90th percentile (p90), mean, median, minimum, maximum, skewness, kurtosis, and entropy. The independent‐sample *t* test, linear correlation analysis, receiver operating characteristics (ROC) curve analysis, Kaplan–Meier analysis, and Cox proportional hazard regression were performed.

**Results**: *MGMT* promoter methylation and *TERT* promoter mutation were detected in 53.2% and 44.3% of GBM patients, respectively. The ADC_p10_ in MGMT promoter unmethylated group was significantly lower than that in the MGMT promoter methylated group (*p *= 0.005). There were significant differences in ADC_min_, ADC_p10_, ADC_mean_, and entropy between TERT promoter mutant and wild‐type groups. Entropy showed the best diagnostic performance in differentiating between positive and negative *TERT* groups (AUC = 0.722, *p *= 0.001). Overall survival (OS) showed a positive correlation with ADC_min_. The *TERT* promoter mutation was the only independent prognostic factor for GBM.

**Conclusions**: ADC histogram analysis may be a potential noninvasive biomarker for differentiating MGMT and TERT molecular markers and providing prognostic information for GBM patients.

## Graphical abstract

Personalized treatment based on molecular markers may help improve the prognosis of glioblastoma (GBM). We aimed to evaluate whether apparent diffusion coefficient (ADC) histogram analysis can better predict MGMT and TERT molecular characteristics and to determine the prognostic relevance of genetic profile in patients with GBM.

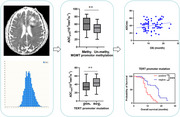

## 1 Introduction

Glioblastoma (GBM) is the most prevalent primary malignant tumor of the central nervous system in adults and is characterized by high heterogeneity and aggressiveness (van Dijken et al. 2018; Han et al. 2018). Recurrent and progressive disease following the standard treatment of maximal tumor resection followed by adjuvant radiotherapy and concomitant chemotherapy is frequently observed (Stupp et al. 2005). Molecular markers have emerged as a promising approach for personalized treatment and can provide valuable prognostic information in patients with GBM (Papavassiliou and Papavassiliou 2022). Recent studies have shown that certain molecular subtypes of GBM are associated with good prognosis (Mansouri et al. 2019; Song et al. 2022; Ramos‐Fresnedo et al. 2022), leading in part to a shift in the traditional standard treatment model toward individualized treatment for improving survival outcomes.

MGMT promoter methylation and TERT promoter mutation (C228T and C250T) are the most common genetic phenomena associated with prognosis of adult GBM, accounting for 66.42%–80% (Powter et al. 2021; Killela et al. 2013) and 38.49%–73.6% (Killela et al. 2013; Kanas et al. 2017), respectively, in previous reports. MGMT is an important DNA repair enzyme responsible for the specific removal of alkyl adjuncts at the guanine O6 site, thereby effectively preventing the formation of harmful DNA cross‐linking and mutagenic events (Sarkaria et al. 2008). The occurrence of MGMT promoter methylation is associated with significantly reduced MGMT protein expression levels, thus reversing DNA damage induced by alkylating agents such as temozolomide (TMZ) and nitroso urea compounds (Santivasi and Xia 2014; Hegi et al. 2005; Ahmed et al. 2015). This phenomenon eventually causes tumors to become resistant to the TMZ chemotherapy regimen. TERT is implicated in the activation of telomerase, which safeguards the integrity of telomeres located at the terminal ends of chromosomes, thereby facilitating uninterrupted cellular division and proliferation in cancer cells (Arita et al. 2016; Olympios et al. 2021). Several studies propose that mutations in the TERT promoter may synergize with other oncogenic mutations, hastening the progression of GBM (Arita et al. 2013; Zhang et al. 2020). Despite their prevalence in GBM cases, there remains a dearth of comprehensive understanding regarding the interplay and prognostic significance between MGMT and TERT markers. Consequently, further investigation into the role of MGMT and TERT markers in GBM is warranted.

In clinical practice, diffusion‐weighted imaging (DWI) has been shown to provide valuable information about the tumor microstructure, cellularity, and heterogeneity. Currently, there is a growing interest in the use of apparent diffusion coefficient (ADC) histogram analysis in various types of tumors. Hu et al. (2017) analyzed the ADC histogram parameters of 57 patients with hepatocellular carcinoma. They found that 5th percentile (p5), 25th percentile (p25), 75th percentile (p75), mean, and median ADC values demonstrated significant differences between low and high Ki‐67 groups and were negatively correlated with Ki‐67 expression. Sun et al. (2020) compared 72 patients with esophageal squamous cell carcinoma examined by dynamic contrast–enhanced magnetic resonance imaging (DCE MRI) before and after chemoradiotherapy (CR); the results showed that the pretreatment median, mean, 10th percentile (p10), and 90th percentile (p90) values of K^trans^ were significantly higher in CR responders than in the non‐CR responders. Similar promising results have also been reported in other types of tumors such as osteosarcoma (Foroutan et al. 2013), metastatic tumors (Kamimura et al. 2019), and gliomas (G. Gihr et al. 2022). In each case, ADC histogram analysis has provided insights into tumor characteristics and treatment outcomes.

Therefore, the purpose of the present study was to determine the differences between MGMT and TERT subtypes based on ADC histograms and to assess their potential prognostic impact in patients with GBM. To the best of our knowledge, there is a paucity of research on TERT in glioma, and our study also aimed to add to the body of knowledge in this area.

## 2 Materials and Methods

### 2.1 Subjects

This study was approved by the local ethics committee. Informed consent was waived from all subjects. One hundred and eight GBM patients with pathologically confirmed GBM were included in the study from two institutions between January 2020 and December 2022. Clinical information such as molecular pathological diagnosis, age, sex, genetic diagnosis results (including *MGMT* and *TERT*), date of diagnosis, date of death, or final follow‐up was collected from the electronic medical records.

The inclusion criteria were as follows: (1) histopathologic and molecular pathological diagnosis consistent with the study, (2) time elapsed between MRI examination and surgery not exceeding 1 week, and (3) patients were examined with 3.0T MRI in order to minimize data variance. The exclusion criteria were as follows: (1) poor image quality affecting the accuracy of diagnosis (*n* = 3), (2) patients with a pathological diagnosis of other types of brain tumors (*n* = 14), and (3) patients without molecular diagnosis (*n* = 12). All patients underwent maximal tumor resection followed by concurrent radiotherapy and adjuvant chemotherapy. Overall survival (OS) was defined as the date from MRI diagnosis to death or the end of follow‐up.

### 2.2 Imaging Acquisition

Imaging data included axial T2WI, T1WI, Gd‐T1WI and DWI were obtained on 3.0T MRI system (Philips, Achieva, Netherlands; GE, Premier, USA; Siemens, Presima, Germany). The MRI parameters are provided in Table S1.

DWI was performed with a single‐shot spin‐echo EPI sequence in the axial plane with *b*‐values of 0 and 1000 s/mm^2^. Each *b* value is diffused in three directions. ADC is a mapping derived from the DWI imaging sequence. Postcontrast T1‐weighted images were taken following intravenous injection of gadoterate meglumine through the median cubital vein at a flow rate of 2 mL/s (0.2 mL/kg body weight).

### 2.3 ADC Map Histogram Analysis

ADC, T2WI, and Gd‐T1WI are exported from our institutional archive in DICOM format via the PACS system. All sequences were processed by 3D slicer (http://www.slicer.org/). Registration and resampling of all these sequences were carried out to generate standardized images with good repeatability and generalization. The specific process was as follows: ADC maps, T2WI, and Gd‐T1WI were loaded into the graphical user interface. The region of interest (ROI) of the entire tumor entity was mapped along the tumor margin on T2WI sequences (tumor without enhancement) or Gd‐T1WI images (tumor with enhancement) and automatically registered on the corresponding ADC map. Finally, the following features of the ADC histogram of the whole tumor volume were computed: p10, p90, mean, median, minimum, maximum, skewness, kurtosis, and entropy. All measurements were taken by two senior radiologists with 10 years of experience in central nervous system diagnosis (T.L. and Y.H.). Interclass correlation coefficient (ICC) between 0.75 and 1 was considered indicative of good agreement. Any disagreement between the two neuroradiologists was resolved by consensus.

### 2.4 Molecular Testing for *IDH*, *MGMT*, and *TERT* Status

Pathological diagnosis was based on the 2021 5th Edition classification criteria for central nervous system brain tumors. *IDH* and *TERT* mutations were detected by next‐generation sequencing, and MGMT methylation was detected by pyrosequencing (PCR), as previously described (Arita et al. 2016; G. Gihr et al. 2022; G. A. Gihr et al. 2020).

### 2.5 Statistical Analysis

SPSS 27.0 software was used for statistical analyses. Sex distribution among the groups was compared using the Mann–Whitney *U* test, whereas the age and ADC histogram parameters were compared using the chi‐square test. Pearson correlation analysis was used to assess the correlation between various parameters. The diagnostic performance of the parameters was assessed using receiver operating characteristic (ROC) curve analysis. Kaplan–Meier analysis and the Cox proportional hazards model were performed for survival analysis. *p* values < 0.05 were considered indicative of statistical significance for all tests.

## 3 Results

**FIGURE 1:**
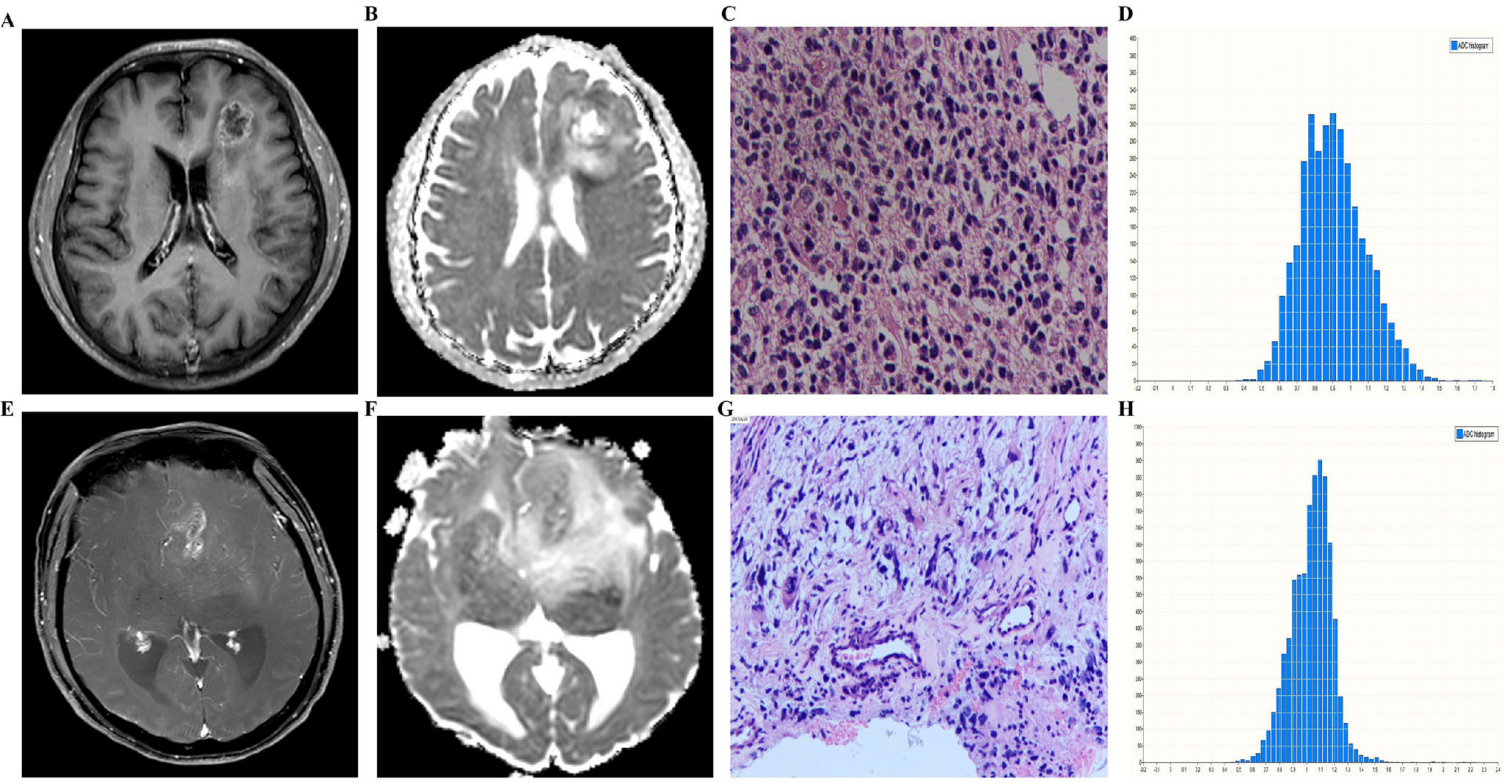
Comparison of whole tumor ADC histograms for GBMs with unmethylated *MGMT* promoter (upper row, Case 1) and methylated *MGMT* promoter (bottom row, Case 2). In Case 1, (A–D) represent tumor Gd‐T1WI, ADC map, the corresponding H&E‐stained sections, and ADC histogram (*x*‐axis: ADC values, *y*‐axis: number of voxels), respectively. There was a ring‐like enhancement of tumor (A) with low signal on ADC map (B) in the solid portion. The ADC value corresponding to the crest of the whole tumor histogram was approximately 0.9 × 10^−3^ mm^2^ s^−1^ (D). In Case 2, (E–H) represent tumor Gd‐T1WI, ADC map, the corresponding H&E‐stained sections, and ADC histogram (*x*‐axis: ADC values, *y*‐axis: number of voxels), respectively. It showed a small patchy enhancement of tumor (E) with local low signal on ADC map in the solid portion (F). The ADC value corresponding to the crest of the whole tumor histogram was approximately 1.1 × 10^−3^ mm^2^ s^−1^ (H). (C) and (G) showed pleomorphic nuclei and abundant cytoplasm in the tumor cells.

**FIGURE 2:**
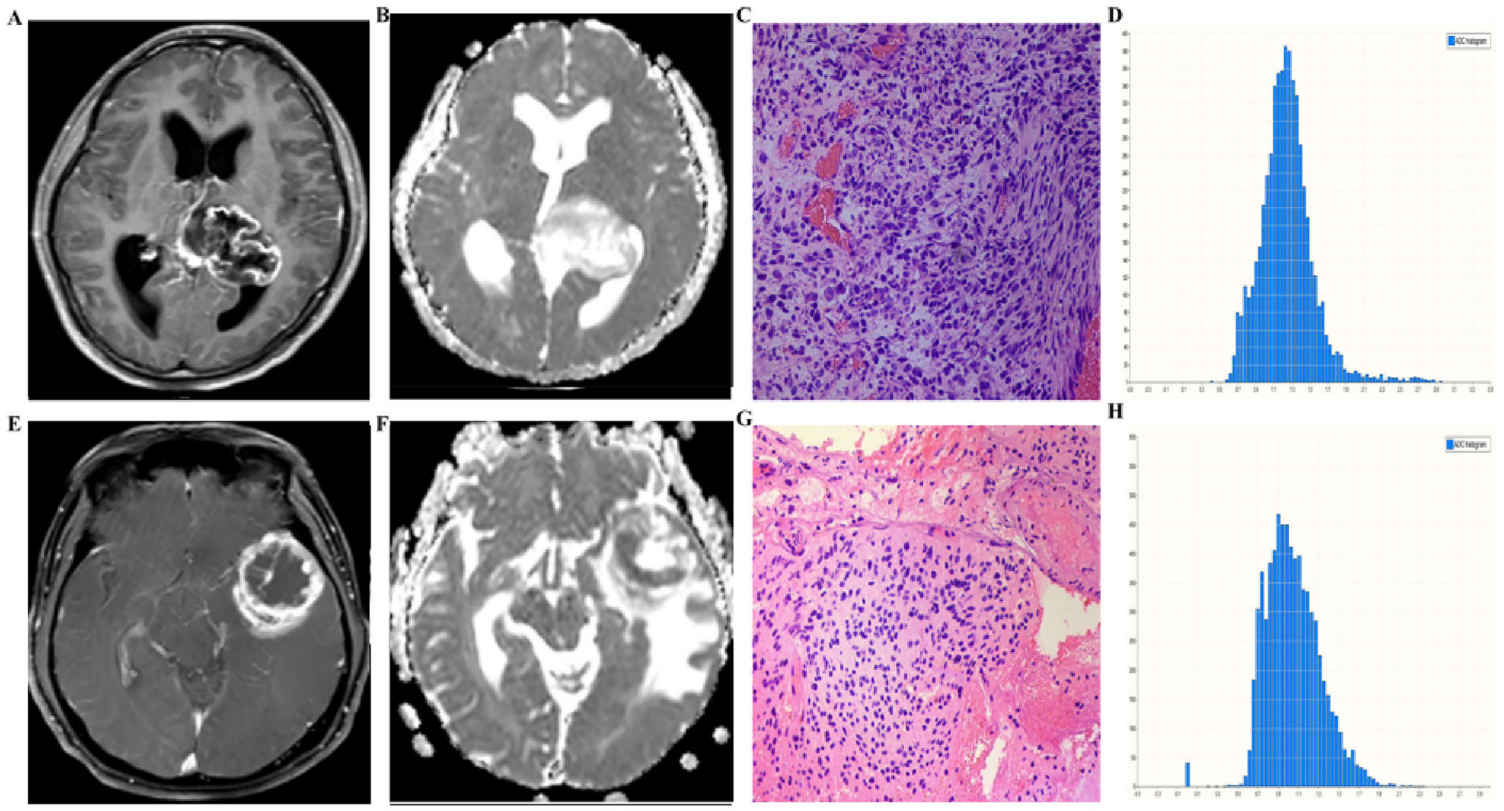
Comparison of the whole tumor ADC histogram for GBMs with an unmutated *TERT* promoter (upper row, Case 1) and a mutated *TERT* promoter (bottom row, Case 2). In Case 1, (A–D) represent tumor Gd‐T1WI, ADC map, the corresponding H&E‐stained sections, and ADC histogram (*x*‐axis: ADC values, *y*‐axis: number of voxels), respectively. It showed a ring‐like enhancement of tumor with medium signal on ADC map (B) in the solid portion. The ADC value corresponding to the crest of the whole tumor histogram was approximately 1.3 × 10^−3^ mm^2^ s^−1^ (D). In Case 2, (E–H) represent tumor Gd‐T1WI, ADC map, the corresponding H&E‐stained sections, and ADC histogram (*x*‐axis: ADC values, *y*‐axis: number of voxels), respectively. Case 2 shows a ring‐like enhancement of tumor (E) with low signal on ADC map (F) in the solid portion. The ADC value corresponding to the crest of the whole tumor histogram was approximately 0.9 × 10^−3^ mm^2^ s^−1^ (H). (C) and (G) showed pleomorphic nuclei in the tumor cells.

A total of 79 patients (37 females, 42 males; mean age: 49.7 ± 12.6 years [range: 38–72]) were included in this study. MGMT promoter methylation (42/79) and TERT promoter mutation (35/79) were detected in 53.2% and 44.3% patients in our cohort, respectively. The basic clinical characteristics of the GBM patients are presented in Table 1. There were no significant differences observed in terms of age and sex distribution between the MGMT and TERT subgroups. However, a statistically significant difference was found in KPS scores between the MGMT and TERT subgroups. Figures 1 and 2 show representative conventional MRI images, ADC histograms, and corresponding H&E‐stained sections for GBM patients with different MGMT and TERT phenotypes. Table 2 shows the histogram results of the whole tumor.

**TABLE 1:** The basic clinical characteristics of GBM patients.

Parameters	MGMT‐methy	MGMT‐unmethy	*p*	TERT‐mt	TERT‐wt	*p*
Age	56.70 ± 12.41	58.69 ± 4.62	0.273	59.37 ± 11.46	56.18 ± 11.93	0.132
Male	19	23		20	22	
Sex			0.614			0.559
Female	23	14		15	22	
KPS	82.67 ± 14.16	66.32 ± 15.36	0.042	71.66 ± 13.39	85.20 ± 15.36	0.037

**TABLE 2:** ADC histogram analysis of the whole tumor.

ADC histogram parameters	Mean ± SD	Minimum	Maximum
ADC_mean,_× 10^−5^ mm^2^ s^−1^	107.30 ± 33.72	54.01	190.70
ADC_median,_ × 10^−5^ mm^2^ s^−1^	98.78 ± 21.82	59.9	177.55
ADC_min,_ × 10^−5^ mm^2^ s^−1^	40.32 ± 12.76	13.0	65.0
ADC_max,_ × 10^−5^ mm^2^ s^−1^	204.17 ± 44.98	119.7	376.8
ADC_p10_, × 10^−5^ mm^2^ s^−1^	54.54 ± 17.21	20.10	86.20
ADC_p90_, × 10^−5^ mm^2^ s^−1^	157.95 ± 27.14	94.81	233.40
Skewness	1.42 ± 0.64	0.15	3.07
Kurtosis	6.08 ± 2.25	1.63	12.14
Entropy	6.47 ± 1.79	4.28	12.75

Observer comparisons were conducted for imaging parameters, and subsequent statistical analysis demonstrated that the ICC value exceeded 0.8, indicating high reliability of these data.

**FIGURE 3:**
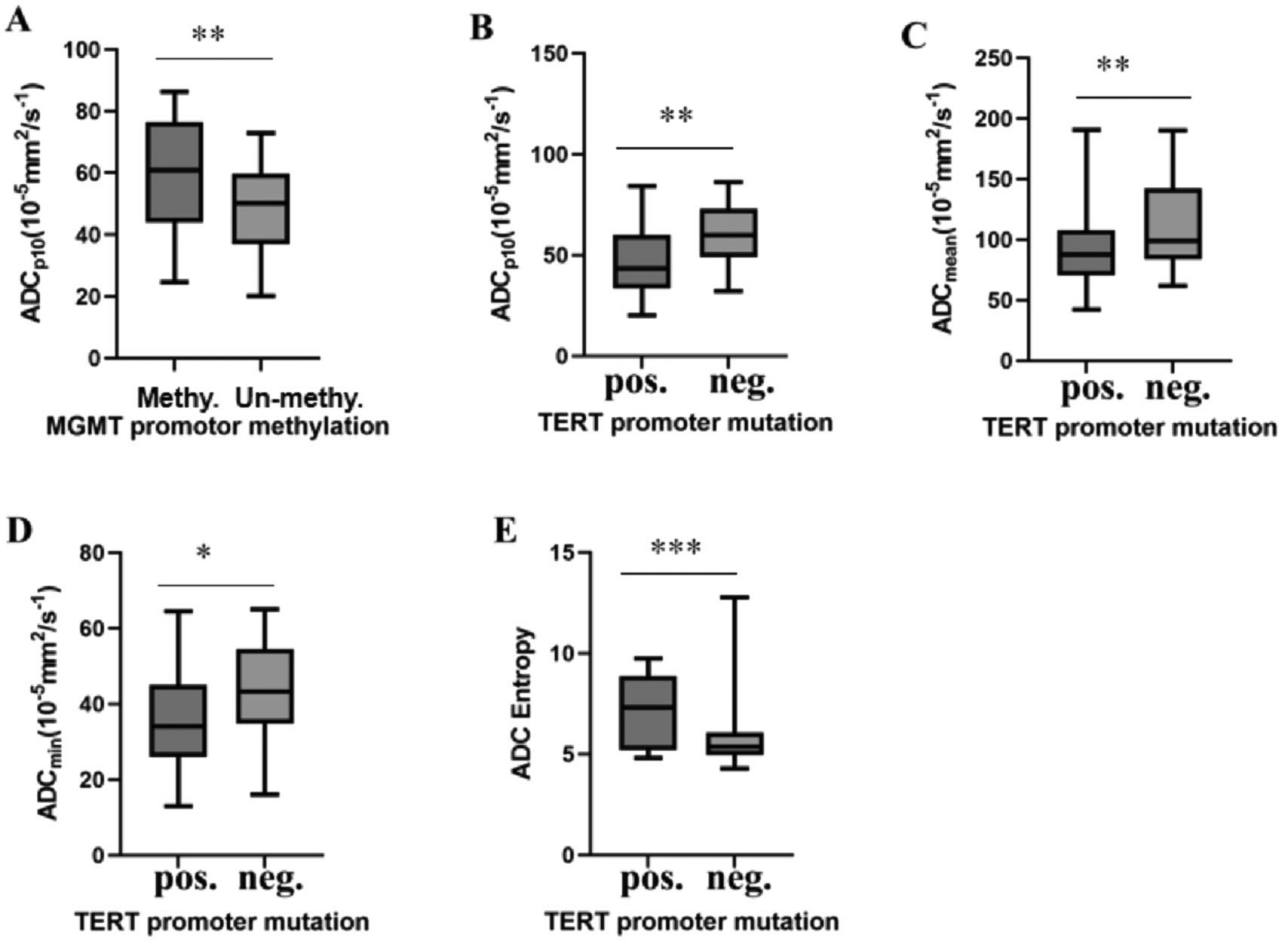
(A–E) show boxplot comparing differences in ADC values between MGMT and TERT subtypes (*p < 0.05, **p < 0.01, ***p < 0.001).

Comparisons of the DWI histogram between the MGMT and TERT subgroups are presented in Tables 3 and 4. The ADC_min_ was significantly lower in the MGMT promoter methylation negative subgroup compared to the MGMT promoter methylation positive subgroup (59.45 ± 18.81 vs. 48.96 ± 13.37, *p *= 0.005). However, there were no significant differences observed among the ADC_p90_, ADC_mean_, skewness, kurtosis, entropy, and the ADC_p10_. There were significant differences in ADC_mean_, ADC_min_, and ADC_p10_ between the TERT promoter positive and negative subgroups (*p *= 0.003, 0.019, and 0.007, respectively). Moreover, the entropy values in the *TERT* mutant group were significantly higher than those in wild‐type group (7.26 ± 1.79 vs. 5.84 ± 1.55, *p *< 0.001). Figure 3A–E shows a boxplot comparing differences in ADC values between MGMT and TERT subtypes.

**TABLE 3:** Comparison of ADC histogram parameters between MGMT promoter methylated and un‐methylated groups.

Parameters	MGMT methylated	MGMT Un‐methylated	*p* values
ADC_mean, _× 10^−5^ mm^2^ s^−1^	98.76 ± 35.77	108.62 ± 36.04	0.228
ADC_median, _× 10^−5^ mm^2^ s^−1^	98.14 ± 28.39	99.50 ± 27.51	0.830
ADC_min, _× 10^−5^ mm^2^ s^−1^	41.52 ± 12.57	38.95 ± 13.01	0.376
ADC_max, _× 10^−5^ mm^2^ s^−1^	205.14 ± 48.73	203.08 ± 40.95	0.840
ADC_p10_, × 10^−5^ mm^2^ s^−1^	59.45 ± 18.81	48.96 ± 13.37	**0.005**
ADC_p90_, × 10^−5^ mm^2^ s^−1^	159.10 ± 26.97	156.64 ± 27.64	0.692
Skewness	1.47 ± 0.56	1.35 ± 0.72	0.400
Kurtosis	6.41 ± 2.52	5.7 ± 1.85	0.154
Entropy	6.40 ± 1.91	6.55 ± 1.68	0.698

*Note*: Significant result is shown in bold.

**TABLE 4:** Comparison of ADC histogram parameters between *TERT* mutant‐type and *TERT* wild‐type groups.

Parameters	TERT mutant‐type	TERT wild‐type	*p* values
ADC_mean, _× 10^−5^ mm^2^ s^−1^	90.34 ± 28.89	113.75 ± 37.99	**0.003**
ADC_median, _× 10^−5^ mm^2^ s^−1^	98.06 ± 24.95	99.35 ± 30.17	0.839
ADC_min, _× 10^−5^ mm^2^ s^−1^	36.56 ± 12.98	43.30 ± 11.90	**0.019**
ADC_max, _× 10^−5^ mm^2^ s^−1^	201.84 ± 40.53	206.03 ± 48.61	0.683
ADC_p10_, × 10^−5^ mm^2^ s^−1^	48.72 ± 18.20	59.17 ± 15.03	**0.007**
ADC_p90_, × 10^−5^ mm^2^ s^−1^	159.65 ± 29.36	156.59 ± 25.50	0.623
Skewness	1.47 ± 0.63	1.37 ± 0.65	0.513
Kurtosis	6.37 ± 2.51	5.85 ± 2.0	0.311
Entropy	7.26 ± 1.79	5.84 ± 1.55	**< 0.001**

*Note*: Significant results are shown in bold.

**FIGURE 4:**
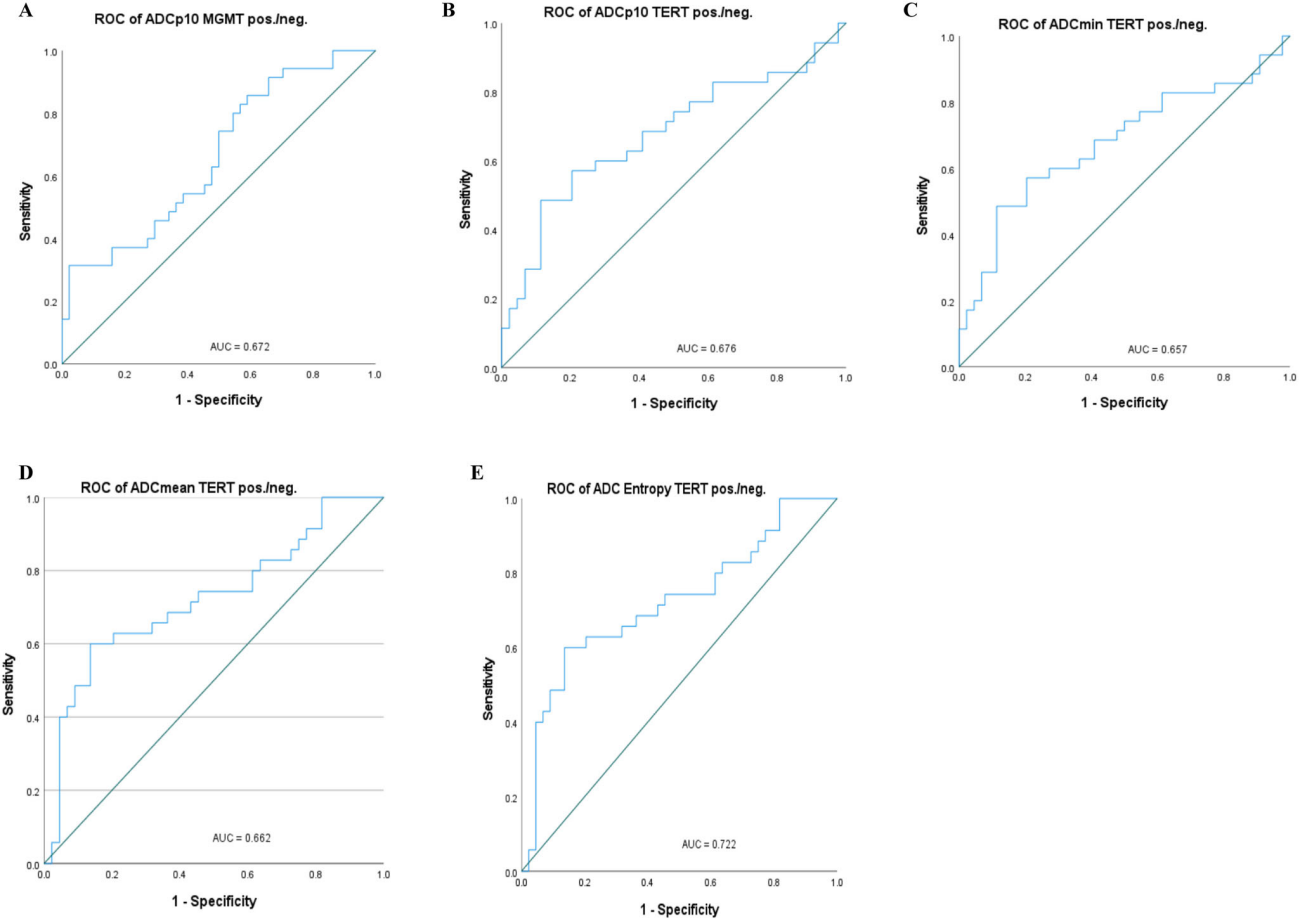
Receiver operating characteristic (ROC) curve analysis of ADC histogram values in *MGMT* promoter methylation and *TERT* mutation groups. (A) stands for the ROC curves for ADCp10 to distinguish MGMT methylated and un‐methylated subtypes; (B‐E) stand for the ROC curves for ADCp10, ADCmean, ADCmin and ADCentropy to distinguish TERT mutant‐type and TERT wlid‐type, respectively.

**FIGURE 5:**
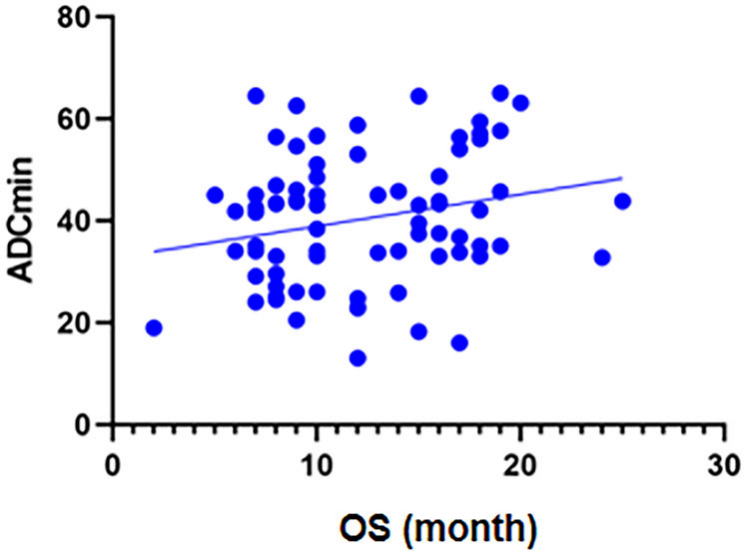
Scatter plot demonstrating the correlation between ADC_min_ and OS in GBM patients (*x*‐axis: OS, *y*‐axis: ADC values). OS shows a positive correlation with ADC_min_.

Figure 4 and Table 5 display the ROC curve analysis results based on ADC histogram values in MGMT promoter methylation and TERT mutation status. Entropy showed the best diagnostic performance in differentiating between positive and negative TERT promoters (area under the curve [AUC]: 0.722; optimal cut‐off value: 5.44; 95% CI 0.605–0.839). In Figure 5, OS showed a positive correlation with ADC_min_ (*R* = 0.260, *p *= 0.020).

**TABLE 5:** ROC curve analysis results between *MGMT* promoter methylation and *TERT* promoter mutation status.

Parameters	AUC	Cutoff	95% CI	*p*
MGMT				
ADC_p10_	0.672	49.55	0.551–0.792	**0.009**
ADC_p90_	0.532	147.24	0.402–0.661	0.627
ADC_min_	0.561	35.90	0.433–0.689	0.066
ADC_max_	0.496	203.40	0.368–0.625	0.957
ADC_mean_	0.408	87.84	0.283–0.533	0.160
ADC_median_	0.47	87.65	0.342–0.598	0.651
Skewness	0.561	1.19	0.432–0.690	0.066
Kurtosis	0.582	5.39	0.454–0.709	0.212
Entropy	0.549	5.85	0.323–0.579	0.455
TERT				
ADC_p10_	0.676	50.11	0.552–0.8	**0.007**
ADC_p90_	0539	145.8	0.409–0.669	0.554
ADC_min_	0.657	34.5	0.533–0.781	**0.017**
ADC_max_	0.469	193.25	0.338–0.599	0.632
ADC_mean_	0.662	89.85	0.542–0.782	**0.014**
Skewness	0.545	1.21	0.417–0.674	0.490
Kurtosis	0.532	5.23	0.401–0.664	0.067
Entropy	0.722	5.44	0.605–0.839	**0.001**

*Note*: Significant results are shown in bold.

The Cox regression analysis showed that TERT promoter mutation was the only independent prognostic factor for GBM. The OS time was 13.5 months, and the survival time in the TERT promoter mutation‐positive group was significantly lower than that in the TERT negative promoter mutation group in this cohort (10.1 months vs. 15.9 months). Kaplan–Meier curve analysis (Figure&amp;#x000A0;6) demonstrated that the OS in the TERT promoter mutation‐positive group was significantly shorter than that in the mutation‐negative group (*p *< 0.001).

**FIGURE 6:**
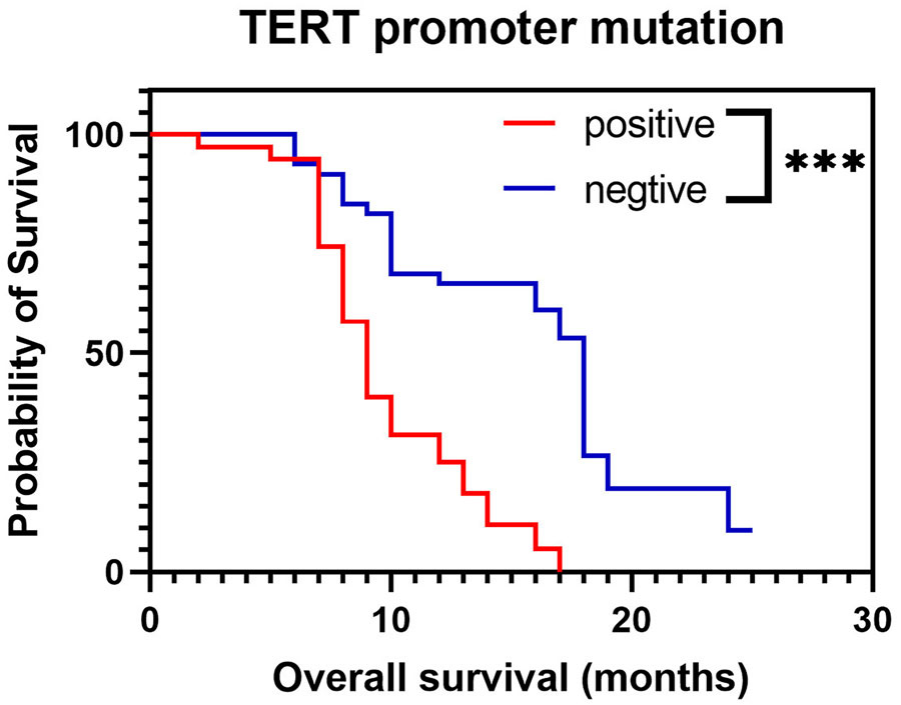
The Kaplan–Meier curves for OS between *TERT* promoter positive and negative groups in GBMs (***p < 0.001).

## 4 Discussion

The heterogeneity of GBM, both at the molecular and histological levels, is a major factor that hinders therapeutic efficacy. Some analyses have demonstrated the association of MGMT and TERT subtypes with clinical outcomes (Romano et al. 2013; Shu et al. 2018). However, the results have been inconsistent, and there is a need for further studies. In this study, we focused on GBM, which is known to be associated with poor prognosis and resistance to treatment. An ADC histogram was performed to analyze the relationship between MGMT and TERT characterization and survival benefits.

In this study, the ADC_p10_ value of the MGMT promoter in the unmethylated group was significantly lower than that in the methylated group. Similar results were reported by Zhang et al. (2022), Moon et al. (2012), and (G. Gihr et al. 2022). These studies suggested that tumors associated with nonmethylation MGMT promoters may have greater heterogeneity or cell density. We also observed that ADC_mean_, ADC_min_, ADC_p10_, and entropy were significantly different between mutated and nonmutated TERT subgroups. *TERT* mutations may lead to a decrease in ADC value by affecting the metabolic activity and molecular diffusion ability of tumor cells (Nguyen et al. 2017). However, some studies found no significant difference in ADC values between TERT‐mutated and nonmutated gliomas (Zhang et al. 2020; Liu et al. 2022; Yamashita et al. 2019; Ivanidze et al. 2019). In these studies, only a small amount of information on the largest level of tumors by ROI measurement was obtained, which may have affected the overall results. To date, there is a paucity of research on ADC and *TERT* mutations in GBMs. Therefore, this mechanism still needs further research and validation to better understand the role of *TERT* mutations in tumor development.

In our study, the OS in the TERT promoter mutation‐positive group was significantly shorter than that in the mutation‐negative group (*p *< 0.001). The Cox proportional risk regression model further confirmed this finding, as the TERT promoter mutation was found to be an independent predictor of shorter OS in GBM patients. Patients with tumor *TERT* mutations in our research had a worse prognosis than those without mutations (mean OS: 10.1 vs. 15.9 months, respectively), corresponding to a 43% reduction in OS time. Some previous studies have demonstrated that mutant TERT promoter is associated with poor OS and progression‐free survival (PFS) in GBM patients (Malkki 2014; Zeng et al. 2020; Eckel‐Passow et al. 2015; Kikuchi et al. 2020). Our results further support the survival disadvantage associated with TERT promoter mutations in GBM patients. Furthermore, OS showed positive correlation with ADC_min_. The results show that ADC value can be an effective prognostic marker in GBM patients.

However, in our study, MGMT promoter methylation, age, sex, or ADC value was not found to be independent prognostic factors for GBM. Some previous studies analyzing the prognostic relevance of MGMT status in GBMs have yielded inconsistent results. In a retrospective study of 47 patients with GBM by Romano et al. (2013), MGMT was not found to be an independent prognostic factor, although the methylated group showed better OS and PFS than the unmethylated group. Shu et al. (2018) investigated 304 GBM patients, and they identified age and MGMT as independent prognostic factors; in addition, combining TERT and MGMT with other factors produced different survival benefits. Further studies are required to elucidate the underlying difference.

On ROC curve analysis, entropy had the highest AUC value (0.722) among all the parameters, indicating that entropy can be used as a potential biomarker for identifying *TERT* mutations in GBMs. G. Gihr et al. (2022) also reported that entropy was the only indicator that distinguished *IDH* mutations from wild‐type ones in low‐grade glioma. Entropy is a measure of the randomness or disorder in the pixel intensity distribution of an image (Just 2014). In this study, the entropy values reflect the heterogeneity of the ADC maps, which is associated with the degree of cellularity and microstructural complexity of the tumors. The higher the entropy value, the greater is the heterogeneity of the tumor tissue. Therefore, the results of this study demonstrate the potential of entropy as a useful marker and provide insights into the complex nature of tumor heterogeneity.

Some limitations of this study should be considered while interpreting the results. First, this was a retrospective analysis of data from only two centers. Prospective, multicenter research should support more robust conclusions. Furthermore, despite having the best ability to distinguish *TERT* mutation types when compared to other parameters, the AUC was only 0.722. Integrating DWI with other advanced quantitative MRI techniques and radiomics may help increase the diagnostic efficiency. Last, although the images were standardized, the parameters of different manufacturers may still cause deviation in the results. Prospective studies are required to further standardize the scan sequences and parameters in order to minimize bias.

In conclusion, ADC histogram analysis can provide valuable insights into the MGMT and TERT molecular characterization of patients with GBM and further provide valuable prognostic information. Therefore, ADC histogram analysis may be recommended for preoperative MRI diagnosis, especially for tumors with overlapping conventional MRI findings.

## Author Contributions

L.C. and R.L. have made a substantial contribution to the concept or design of the article, the acquisition, analysis, interpretation of data for the article, and drafted the article. M.W., C.Y.T., and R.L. revised article critically for important intellectual content. L.F.T. and L.Z.H. collected the data of all patients and made an analysis and interpretation of data for the article. L.Z. and T.L. have agreed to be accountable for all aspects of the work in ensuring that questions related to the accuracy or integrity of any part of the work are appropriately investigated and resolved. All authors contributed to the article and approved the submitted version.

## Ethics Statement

All procedures performed in studies involving human participants were in accordance with the ethical standards of the institutional and/or national research committee and with the 1964 Helsinki Declaration and its later amendments or comparable ethical standards.

## Consent

The informed consent was obtained from all subjects.

## Conflicts of Interest

The authors declare no conflicts of interest.

### Peer Review

The peer review history for this article is available at https://publons.com/publon/10.1002/brb3.70175.

## Supporting information



Supplementary Table 1. Magnetic resonance imaging protocol.

## Data Availability

The data that support the findings of this study are available from the corresponding author upon reasonable request.
