# Supplementary material for: Assessment of MGMT and TERT Subtypes and Prognosis of Glioblastoma by Whole Tumor Apparent Diffusion Coefficient Histogram Analysis
Source: Brain Behav. 2024 Dec 31;15(1):e70175. doi: 10.1002/brb3.70175 (PMC11686090; doi:10.1002/brb3.70175)
Supplement: Supplementary file 1 — Supplementary Table 1. Magnetic resonance imaging protocol. [file BRB3-15-e70175-s001.doc]

**The MRI parameters are provided in Supplementary Table 1.**

**Supplementary Table 1.** Magnetic Resonance Imaging Protocol

|  | **3.0 T Philips**  **(n=37)** | **3.0 T Siemens**  **(n=11)** | **3.0 T GE**  **(n=26)** |
| --- | --- | --- | --- |
| **T2WI** | | | |
| TR/TE (ms) | 2600/80 | 4950/104 | 5844/129 |
| FOV | 512*512 | 378*448 | 512*512 |
| FA (^。^) | 90 | 90 | 90 |
| Matrix | 256*217 | 256*203 | 256*217 |
| Slice thickness/gap (mm) | 5/3 | 5/2.5 | 5/2.5 |
| **T1WI** | | | |
| TR/TE (ms) | 1000/15 | 1800/25 | 1500/20 |
| FOV | 512*512 | 512*512 | 512*512 |
| FA (^。^) | 90 | 90 | 90 |
| Matrix | 256*217 | 256*203 | 256*217 |
| Slice thickness/gap (mm) | 5/3 | 5/2.5 | 5/2.5 |
| **DWI** | | | |
| TR/TE (ms) | 2429/81 | 4850/81 | 2155/61.2 |
| FOV | 256*256 | 196*196 | 256*256 |
| FA (^。^) | 90 | 90 | 90 |
| Matrix | 120*127 | 256*203 | 256*217 |
| Slice thickness/gap (mm) | 5/1 | 5/1 | 5/1 |
| MGMT-methy (n=42) | 16 | 12 | 14 |
| TERT-mutant (n=35) | 13 | 14 | 8 |
